# The Impact of Insulin-Induced Lipodystrophy on Glycemic Variability in Pediatric Patients with Type 1 Diabetes

**DOI:** 10.3390/children9071087

**Published:** 2022-07-20

**Authors:** Fortunato Lombardo, Bruno Bombaci, Angela Alibrandi, Giulia Visalli, Giuseppina Salzano, Stefano Passanisi

**Affiliations:** 1 Department of Human Pathology in Adult and Developmental Age “Gaetano Barresi”, University of Messina, Via Consolare Valeria 1, 98124 Messina, Italy; fortunato.lombardo@unime.it (F.L.); brunobombaci@gmail.com (B.B.); giulia.vsl@hotmail.com (G.V.); gsalzano@unime.it (G.S.); 2 Unit of Statistical and Mathematical Sciences, Department of Economics, University of Messina, 98122 Messina, Italy; aalibrandi@unime.it

**Keywords:** coefficient of variation, glycemic control, lipohypertrophy, lipoatrophy, prevalence

## Abstract

Lipodystrophy is the most common dermatological complication in patients with diabetes on insulin therapy. Despite the high frequency of lipodystrophy, there are still several difficulties in giving advice about avoidance into practice among children and adolescents with type 1 diabetes and their caregivers. This cross-sectional study aims to evaluate the prevalence of insulin-induced lipodystrophy in a cohort of pediatric patients with type 1 diabetes, to identify associated clinical factors and to assess its influence on glycemic control. Two hundred and twelve patients attending our Diabetes Center during a three-month period were enrolled. The presence of lipodystrophy was assessed by inspection and palpation procedures. Demographic and clinical data including type of treatment, frequency of rotation of insulin administration sites, and glucose metrics of the previous 30 days were assessed and statistically analyzed. Prevalence of lipohypertrophy was 44.3%. Two patients were affected by lipoatrophy (0.9%). Improper rotation of insulin administration sites and low awareness on lipodystrophy were associated to the occurrence of this skin condition (*p* = 0.050 and *p* = 0.005, respectively). When comparing patients with and without lipodystrophy, a significant difference in glycemic variability parameters was detected (*p* = 0.036 for coefficient of variation, *p* = 0.029 for standard deviation score of glucose levels). Lipodystrophy still represents a common complication in patients on insulin therapy. The present study reveals its negative impact on glycemic variability. This finding emphasizes the importance of prevention strategies to minimize the occurrence of this dermatological complication that may interfere with clinical history of the disease.

## 1. Introduction

Type 1 diabetes (T1D) is a chronic disease that still represents a global public health challenge. Health systems and researchers have been making noteworthy efforts to introduce novel therapeutic tools aimed to optimize glycemic control and reduce the incidence of long-term complications [1]. Over the last few decades, significant advances have been made in the management of patients with T1D, with the advent of intensive insulin treatment regimens [2], the introduction of new types of insulin [3], and technologic devices such as insulin pumps and continuous glucose monitoring systems [4]. Despite numerous benefits in the management of the disease, some barriers still exist. Among these, skin reactions are increasingly reported and may hinder the achievement of optimal glycemic control [5]. Lipodystrophy is the most common skin adverse effect [6]. This condition, affecting subcutaneous adipose tissue, can be classified into lipohypertrophy and lipoatrophy, which are characterized by different pathogenetic mechanisms and prevalence. Lipohypertrophy is more common than lipoatrophy [7] and is characterized by the local accumulation of fat tissue in a subcutaneous insulin injection site. It appears as a thickened, swollen skin area, often associated with increased consistency on palpation [8]. Histological examination reveals the presence of adipocytes with increased size and containing multiple lipid droplets [9]. Pathogenic mechanisms are still uncertain, but repetitive mechanical trauma derived from needle use and local trophic effects of insulin might produce an excessive fat tissue growth [10]. Lipoatrophy is clinically related to the occurrence of a visible cutaneous depression in skin areas used for subcutaneous insulin administration. The pathogenic mechanism is unclear, but immune-mediated damage has been hypothesized [11], and several studies investigating the association with high insulin antibody blood levels [12] and other concurrent autoimmune diseases (i.e., coeliac disease, Hashimoto thyroiditis) have been conducted [13]. Other possible causes are repetitive mechanical trauma, cryoinjury, and abscess formation [14]. Histologically, lipoatrophy is characterized by massive mast cell infiltration. Lipodystrophy may affect insulin absorption in skin areas involved [15,16], resulting in negative impact on blood glucose levels [17,18] and glycemic variability [16]. The aims of our study are to evaluate the prevalence of lipodystrophy among pediatric patients with T1D, to identify associated factors and to assess the influence on glycemic control.

## 2. Materials and Methods

In this cross-sectional study, we recruited children and adolescents with T1D attending the pediatric diabetes outpatient service of our tertiary-care Centre (University Hospital of Messina) from April to July 2021, for routine check-ups. The study was exempt from ethical committee approval since it was confined to anonymized and unidentifiable data routinely collected at our Diabetes Centre. Inclusion criteria were age < 18 years, duration of diabetes > 1 year and subcutaneous insulin therapy for a minimum of 6 months. Exclusion criteria were partial clinical remission according to Hvidovre Study Group definition [19], presence of contact dermatitis or other T1D-related skin disorders different from lipodystrophy, use of steroids or other drugs known to have a relevant impact on glycemic control, and changes in insulin type treatment in the last 6 months. Patients enrolled were assessed for the presence of lipodystrophy by two different evaluators at a single time performing inspection and palpation techniques of the skin areas used for subcutaneous insulin administration. To better identify lipohypertrophy, the palpation method was aligned to the one proposed by Gentile et al. [20], consisting of a combination of accurate, slow, horizontal, and vertical fingertip movements followed by pinching maneuvers. When present, lipodystrophy was classified and recorded as lipoatrophy or lipohypertrophy. Evaluators were not previously informed about the presence of lipodystrophy, insulin administration habits, and grade of awareness of patients and their caregivers. The following demographic and clinical data were collected: age, gender, duration of disease, auxological parameters, type of treatment (multiple daily injections or continuous subcutaneous insulin infusion), total daily insulin dose, last year mean value of glycated hemoglobin (HbA1c), and application of topic creams to treat lipodystrophic lesions. Injection sites (abdomen, buttock, thighs, and arms), number of daily injections, type of insulin, and needle length of patients on multiple daily injections (MDI) were recorded. For patients on continuous subcutaneous insulin infusion (CSII), data regarding brands and models of insulin pump, insertion sites, replacement interval of infusion sets, and type of insulin were collected. Glycemic control of enrolled patients using continuous glucose monitoring (CGM) or flash glucose monitoring (FGM) systems was assessed. The following metrics related to the last 30 days before the appointment were collected: mean and standard deviation score (SDS) of blood glucose levels, time in range (TIR—time expressed in percentage in the ideal range of glucose between 3.9 and 10 mmol/L), time above range (TAR—time expressed in percentage above 10 mmol/L), time below range (TBR—time expressed in percentage below 3.9 mmol/L), and coefficient of variation (CV) expressed in percentage. Only data relating to a daily sensor use >60% were considered for statistical analysis. Finally, patients and their caregivers were asked about their awareness and knowledge on lipodystrophy, their routine on injection site rotation, and about any use of topical medications to prevent or treat lipodystrophic lesions. 

### Statistical Analysis

Numerical data were expressed as mean, standard deviation, median, and interquartile range (Q1–Q3), and the categorical variables as absolute and percentage. The non-parametric approach was used since the numerical variables were not normally distributed, as verified by the Kolmogorov–Smirnov test. In order to identify possible significant differences between patients with and without lipodystrophy, the Mann–Whitney test was applied with reference to numerical parameters and the Chi-Square test with reference to categorical variables. The same analysis was performed in the subpopulation of patients using CGM systems. Univariate and multivariate binary logistic regression models were estimated to identify significant predictors of lipodystrophy (yes or no): in particular, we tested the influence of the following covariates: gender, age, duration of diabetes, body mass index (BMI) Z-score, type of therapy, application of creams and rotation of injection sites. The results were expressed as odds ratio (OR), 95% confidence interval (CI) and *p*-value. In addition, multiple linear regression models were estimated to identify significant predictors of glycemic variability, and the analyzed covariates were lipodystrophy, age, gender, BMI Z-score, and therapy.

Statistical analyses were performed using IBM SPSS for Windows, Version 22 (Armonk, NY, USA, IBM Corp.). A *p*-value < 0.05 was considered to be statistically significant.

## 3. Results

Our study population consisted of a cohort of 212 patients with a mild prevalence of male subjects (58%). Mean age of study participants was 11.9 ± 4.7 years. Duration of disease was 4.8 ± 3.4 years. At the time of the study check-up, the mean BMI Z-score was 0.72 ± 0.96. Ninety-two (43.4%) patients were on MDI therapy, while 120 (56.6%) used insulin pumps. Overall, daily insulin dose was 0.84 ± 0.26 IU/kg. Mean value of HbA1c of the preceding 12 months was 6.8 ± 1.6% (53.9 ± 9.8 mmol/mol). CGM and FGM systems were adopted by 151 (71.2%) study participants. The assessment for lipodystrophic lesions revealed the presence of lipohypertrophy in 94 (44.3%) patients, of which 50 (23.6%) had a single lesion and 44 (20.7%) had multiple lesions. Lipoatrophy was found in 2 (0.9%) patients. Demographic, anthropometric and clinical data of the study participants are summarized in Table 1. The great majority of patients along with their caregivers (90.5%) showed awareness of lipodystrophies, their associated risks, and prevention strategies. 

### 3.1. Associated Factors

Anthropometric and clinical data on two subgroups of patients with and without lipodystrophy are summarized in Table 2. A significant association between male gender and the occurrence of lipodystrophy was found (*p* = 0.02). This finding was confirmed by univariate and multivariate logistic regression models, which revealed a correlation between male gender and lipodystrophy onset, with an almost doubled risk compared to female patients (OR 1.89; 95% CI 1.053–3.394; *p* = 0.033). Lack of awareness on this dermatological issue was associated with the occurrence of lipodystrophy (*p* = 0.005). A significant association between improper rotation of insulin administration areas and presence of lipodystrophy was found (*p* = 0.050). No associations between lipodystrophy and age, duration of disease, BMI Z-score, treatment type, or daily insulin dose were revealed. 

Among patients on MDI therapy, there was a significant association between length of pen needles used and the presence of lipodystrophy (*p* = 0.039). Number of injection sites, daily injections, and insulin type were not associated with lipodystrophy. When considering patients using insulin pumps, we found a significant association between the involvement of ≥2 insertion areas and the presence of lipodystrophy (*p* = 0.024). No associations between lipodystrophy and other treatment-related factors including device type, replacement interval of infusion sets, and insulin type were found.

### 3.2. Glycemic Control

**Figure 1:**
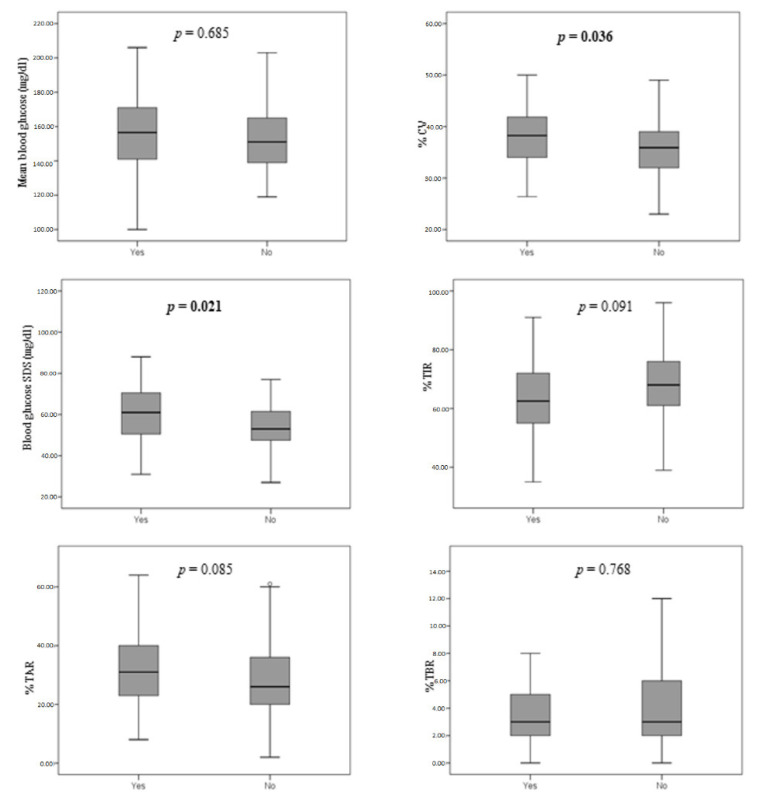
Boxplots illustrating the comparison of CGM data among patients according to the presence of lipodystrophy.

Data on the glycemic control of patients using CGM or FGM systems are summarized in Figure 1.

Comparing patients with and without lipodystrophy, a difference in CV (*p* = 0.036) and blood glucose SDS (*p* = 0.021) was detected. This finding was confirmed by a linear regression model, which showed a direct influence of lipodystrophy on CV (B = 2.050; 95% CI 0.062–4.161; *p* = 0.027). Finally, the age-adjusted linear regression model further confirmed a negative impact of lipodystrophy on CV, which was more evident in younger patients (Table 3). Conversely, other data including TIR, TAR, TBR, mean blood glucose, and last 12-month mean HbA1c were not influenced by the presence of lipodystrophy.

## 4. Discussion

In our study, lipohypertrophy was present in 44.3% of children and adolescents with T1D. This finding is consistent with similar studies on pediatric patients, which showed a prevalence ranging from 37.5% to 56.8% [17,21,22,23]. Similarly, a recent meta-analysis, which reviewed studies on both pediatric and adult patients, found a prevalence of lipohypertrophy of 38% [24]. This rate appears to be slightly higher when considering only pediatric populations [12,25]. Frequency of lipohypertrophy was similar in study participants on MDI therapy and in those using insulin pumps, in accordance with previous literature data [21,26]. 

Since the introduction of modern insulin analogues, the frequency of lipoatrophy has drastically decreased [27]. The low prevalence revealed in our study reflects previous literature data [23,28,29]. Our patients that presented with lipoatrophy were on CSII therapy. Interestingly, a new rise of lipoatrophy incidence has recently been reported among children using insulin pumps [30,31]. 

Our findings confirm that failure to rotate insulin injection sites is closely related to the occurrence of lipodystrophy, as already demonstrated by previous studies [21,32,33]. The latest International Society for Pediatric and Adolescent Diabetes (ISPAD) clinical practice consensus guidelines on insulin therapy, including recommendations on administration techniques, highlighted the paramount role of proper site rotations to prevent dermatological complications [34]. Although patients attending our Diabetes Centre and their caregivers are routinely educated to rotate insulin administration sites, some subjects disregard this clinical recommendation, resulting in high susceptibility to onset of lipodystrophy. 

According to our results, male patients present a higher risk of lipodystrophy. To our knowledge, this evidence has previously been described in only one study [22], making its significance uncertain and worthy of further investigations. Conversely, other patients’ demographical and clinical features such as younger age, lower BMI, and higher daily insulin dose, which have been demonstrated as factors associated to increased lipodystrophy risk in other studies [22,26,35,36], did not influence the occurrence of lipodystrophy in our study population.

Several experimental studies have identified lipodystrophy as a cause of impaired insulin absorption [8,15,16,37]. Johansson et al. investigated differences between plasma insulin profiles in patients after injections on lipohypertrophic tissue and on intact skin, and found lower Cmax after administration through affected skin [37]. Another study found a reduction in insulin absorption and a marked recurrence of postprandial glucose excursions after injections on lipohypertrophic skin [16]. Some authors evaluated the influence of lipodystrophy on glycemic control, by using HbA1c and hypoglycemic events as principal outcomes [6,26,33,36,38]. However, findings from these studies are controversial and no clear association between lipodystrophy and the above-mentioned parameters has been shown. To the best of our knowledge, this is the first study that considers CGM metrics to assess the influence of lipodystrophy on glycemic control. No association between lipodystrophy presence and variations of CGM metrics such as mean blood glucose, TIR, TAR, and TBR was detected. These parameters, similar to HbA1c, are directly related to average glycemic levels. Conversely, CV and blood glucose SDS, which are useful clinical targets to evaluate individual glycemic variability, significantly vary in patients with lipodystrophy. Specifically, we can assume that the presence of lipodystrophic lesions is related to an increase in frequency and range of glycemic excursions due to alteration of insulin absorption patterns. This results in a relevant negative effect on short-term glycemic variability of patients with T1D. It is well-known that glycemic variability represents an independent risk factor for micro- and macrovascular diabetic complications [39,40]. This evidence emphasizes the importance of a complete awareness of patients and their caregivers about this diabetes-related dermatological complication, as it markedly interferes with the clinical history of the disease. The influence of lipodystrophy on glycemic variability appeared more evident in younger patients. This finding might be explained by the reduced skin surface available for insulin administration in these patients.

## 5. Conclusions

Despite recent advances in the management of diabetes and the rise of new technologies for insulin administration, lipodystrophy still represents a troublesome complication for children and adolescents suffering from T1D. The negative impact on glycemic variability revealed by our study highlights the need of more efficient strategies for its prevention, emphasizing the concept of proper insulin administration procedures at each outpatient ambulatory.

## Figures and Tables

**Table 1:** Descriptive statistics for categorical (percentages) and numerical (mean ± SDS and interquartile ranges) variables of the 212 patients included in study.

Variables	Percentage and Mean ± SDS	Median (IQR)
**Age (years)**	11.9 ± 4.7	12.9 (9.1; 15.7)
**Gender** Male Female	123 (58%) 89 (42%)	
**Duration of diabetes (years)**	4.8 ± 3.4	4 (2; 7)
**BMI Z-score**	0.72 ± 0.96	0.78 (0.18; 1.39)
**Insulin treatment type** Multiple daily injections Insulin pump	92 (43.4%) 120 (56.6%)	
**Daily insulin dose (IU/kg/die)**	0.84 ± 0.26	0.82 (0.70; 1.00)
**Last year mean value HbA1c (%)**	6.8 ± 1.6	7.0 (6.5; 7.5)
**Last year mean value HbA1c (mmol/mol)**	53.9 ± 9.8	53 (49; 58)
**Glucose monitoring system** Self-monitoring of blood glucose Flash or continuous glucose monitoring	61 (28.8%) 151 (71.2%)	
**Presence of lipodystrophy** Lipohypertrophy *Single* *Multiple* LipoatrophyNone	94 (44.3%) *50 (23.6%)* *44 (20.7%)* 2 (0.9%) 116 (54.7%)	
**Awareness of the problem** Yes No	191 (90.5%) 20 (9.5%)	

BMI: Body Mass Index; HbA1c: glycated hemoglobin.

**Table 2:** Comparison of anthropometric and clinical data among patients with and without lipodystrophy.

	Lipodystrophy	No Lipodystrophy	*p*-Value
**Number of patients**	96	116	
**Gender** Male Female	64 (66.7%) 32 (33.3%)	59 (50.9%) 57 (49.1%)	0.020
**Age (years)**	11.8 ± 4.8	12.1 ± 4.6	0.673
**Duration of diabetes (years)**	5.2 ± 3.6	4.5 ± 3.2	0.215
**BMI z-score**	0.62 ± 0.94	0.80 ± 0.96	0.165
**Insulin treatment type** Multiple daily injections Insulin pump	47 (49.0%) 49 (51.0%)	45 (38.8%) 71 (61.2%)	0.137
**Rotation of injection/insertion sites** Yes No	77 (80.2%) 19 (19.8%)	104 (89.7%) 12 (10.3%)	0.050
**Awareness of the problem** Yes No	81 (84.4%) 15 (15.6%)	110 (95.7%) 5 (4.3%)	0.005
**Creams application** Yes No	35 (36.5%) 61 (63.5%)	34 (29.3%) 82 (70.7%)	0.269
**Daily insulin dose (IU/kg/die)**	0.88 ± 0.25	0.81 ± 0.27	0.085
**Last year mean value HbA1c (%)**	6.8 ± 1.7	6.9 ± 1.5	0.397
**Last year mean value HbA1c (mmol/mol)**	53.8 ± 9.3	53.9 ± 10.3	

BMI: Body Mass Index; HbA1c: glycated hemoglobin. Significant p-values are marked in bold.

**Table 3:** Multiple linear regression models for the coefficient of variation.

Variables	B	95% CI	*p*-Value
Lipodystrophy	2.050	0.062–4.161	**0.027**
Lipodystrophy	2.113	0.026-4.199	**0.047**
Age	−0.226	−0.441-0.012	**0.039**
Lipodystrophy	2.208	0.118-4.297	**0.039**
Age	−0.225	−0.439-0.011	**0.039**
Gender	1.262	−0.812–3.337	0.231
Lipodystrophy	1.959	−0.164–4.083	0.070
Age	−0.206	−0.424–0.011	0.063
BMI Z-score	0.024	−1.033–1.081	0.964
Lipodystrophy	1.953	−0.157–4.063	0.069
Age	−0.158	−0.380–0.064	0.162
BMI Z-score	−0.134	−1.192–0.924	0.803
Gender	1.455	−0.649–3.560	0.174
Type of therapy	−2.229	−4.694–0.235	0.076

BMI: Body Mass Index; CI: confidence interval; Significant *p*-values are marked in bold.

## Data Availability

The data that support the findings of this study are not publicly available due to privacy reasons of research participants, but are available from the corresponding author upon reasonable request.

## References

[1] American Diabetes Association (2021). 2. Classification and Diagnosis of Diabetes: Standards of Medical Care in Diabetes-2021. Diabetes Care.

[2] Nathan D.M., Genuth S., Lachin J., Cleary P., Crofford O., Davis M., Rand L., Siebert C. (1993). The effect of intensive treatment of diabetes on the development and progression of long-term complications in insulin-dependent diabetes mellitus. N. Engl. J. Med..

[3] Cefalu W.T., Rosenstock J., LeRoith D., Riddle M.C. (2015). Insulin’s Role in Diabetes Management: After 90 Years, Still Considered the Essential Black Dress. Diabetes Care.

[4] Ferrito L., Passanisi S., Bonfanti R., Cherubini V., Minuto N., Schiaffini R., Scaramuzza A. (2021). Efficacy of advanced hybrid closed loop systems for the management of type 1 diabetes in children. Minerva Pediatr..

[5] Lombardo F., Salzano G., Crisafulli G., Panasiti I., Alibrandi A., Messina M.F., Pajno G.B., Caminiti L., Passanisi S. (2020). Allergic contact dermatitis in pediatric patients with type 1 diabetes: An emerging issue. Diabetes Res. Clin. Pract..

[6] Hauner H., Stockamp B., Haastert B. (1996). Prevalence of lipohypertrophy in insulin-treated diabetic patients and predisposing factors. Exp. Clin. Endocrinol. Diabetes.

[7] Wang K., Zhang S., Liu C., Chen Y. (2021). A meta-analysis and meta-regression on the prevalence of lipohypertrophy in diabetic patients on insulin therapy. Therapies.

[8] Heinemann L. (2010). Insulin absorption from lipodystrophic areas: A (neglected) source of trouble for insulin therapy?. J. Diabetes Sci. Technol..

[9] Fujikura J., Fujimoto M., Yasue S., Noguchi M., Masuzaki H., Hosoda K., Tachibana T., Sugihara H., Nakao K. (2005). Insulin-induced lipohypertrophy: Report of a case with histopathology. Endocr. J..

[10] Renold A.E., Marble A., Fawcett D.W. (1950). Action of insulin on deposition of glycogen and storage of fat in adipose tissue. Endocrinology.

[11] Babiker A., Datta V. (2011). Lipoatrophy with insulin analogues in type I diabetes. Arch. Dis. Child..

[12] Raile K., Noelle V., Landgraf R., Schwarz H.P. (2001). Insulin antibodies are associated with lipoatrophy but also with lipohypertrophy in children and adolescents with type 1 diabetes. Exp. Clin. Endocrinol. Diabetes.

[13] Salgin B., Meissner T., Beyer P., Haberland H., Borkenstein M., Fussenegger J., Brand U., Hauffa B.P., Hungele A., Holl R.W. (2013). Lipoatrophy is associated with an increased risk of Hashimoto’s thyroiditis and coeliac disease in female patients with type 1 diabetes. Horm. Res. Paediatr..

[14] Lopez X., Castells M., Ricker A., Velazquez E.F., Mun E., Goldfine A.B. (2008). Human insulin analog—Induced lipoatrophy. Diabetes Care.

[15] Young R.J., Hannan W.J., Frier B.M., Steel J.M., Duncan L.J. (1984). Diabetic lipohypertrophy delays insulin absorption. Diabetes Care.

[16] Famulla S., Hövelmann U., Fischer A., Coester H.V., Hermanski L., Kaltheuner M., Kaltheuner L., Heinemann L., Heise T., Hirsch L. (2016). Insulin Injection into Lipohypertrophic Tissue: Blunted and More Variable Insulin Absorption and Action and Impaired Postprandial Glucose Control. Diabetes Care.

[17] Kordonouri O., Lauterborn R., Deiss D. (2002). Lipohypertrophy in young patients with type 1 diabetes. Diabetes Care.

[18] Chowdhury T.A., Escudier V. (2003). Poor glycaemic control caused by insulin induced lipohypertrophy. BMJ.

[19] Mortensen H.B., Hougaard P., Swift P., Hansen L., Holl R.W., Hoey H., Bjoerndalen H., De Beaufort C., Chiarelli F., Danne T. (2009). New definition for the partial remission period in children and adolescents with type 1 diabetes. Diabetes Care.

[20] Gentile S., Guarino G., Giancaterini A., Guida P., Strollo F., AMD-OSDI Italian Injection Technique Study Group (2016). A suitable palpation technique allows to identify skin lipohypertrophic lesions in insulin-treated people with diabetes. SpringerPlus.

[21] Deeb A., Abdelrahman L., Tomy M., Suliman S., Akle M., Smith M., Strauss K. (2019). Impact of Insulin Injection and Infusion Routines on Lipohypertrophy and Glycemic Control in Children and Adults with Diabetes. Diabetes Ther..

[22] Omar M.A., El-Kafoury A.A., El-Araby R.I. (2011). Lipohypertrophy in children and adolescents with type 1 diabetes and the associated factors. BMC Res. Notes.

[23] Tsadik A.G., Atey T.M., Nedi T., Fantahun B., Feyissa M. (2018). Effect of Insulin-Induced Lipodystrophy on Glycemic Control among Children and Adolescents with Diabetes in Tikur Anbessa Specialized Hospital, Addis Ababa, Ethiopia. J. Diabetes Res..

[24] Deng N., Zhang X., Zhao F., Wang Y., He H. (2018). Prevalence of lipohypertrophy in insulin-treated diabetes patients: A systematic review and meta-analysis. J. Diabetes Investig..

[25] Frid A.H., Hirsch L.J., Menchior A.R., Morel D.R., Strauss K.W. (2016). Worldwide Injection Technique Questionnaire Study: Injecting Complications and the Role of the Professional. Mayo Clin. Proc..

[26] Conwell L.S., Pope E., Artiles A.M., Mohanta A., Daneman A., Daneman D. (2008). Dermatological complications of continuous subcutaneous insulin infusion in children and adolescents. J. Pediatr..

[27] Passanisi S., Salzano G., Lombardo F. (2022). Skin involvement in paediatric patients with type 1 diabetes. Curr. Diabetes Rev..

[28] Richardson T., Kerr D. (2003). Skin-related complications of insulin therapy: Epidemiology and emerging management strategies. Am. J. Clin. Dermatol..

[29] Radermecker R.P., Piérard G.E., Scheen A.J. (2007). Lipodystrophy reactions to insulin: Effects of continuous insulin infusion and new insulin analogs. Am. J. Clin. Dermatol..

[30] Kordonouri O., Biester T., Schnell K., Hartmann R., Tsioli C., Fath M., Datz N., Danne T. (2015). Lipoatrophy in children with type 1 diabetes: An increasing incidence?. J. Diabetes Sci. Technol..

[31] Rosenbloom A.L. (2014). Insulin injection lipoatrophy recidivus. Pediatr. Diabetes.

[32] Pozzuoli G.M., Laudato M., Barone M., Crisci F., Pozzuoli B. (2018). Errors in insulin treatment management and risk of lipohypertrophy. Acta Diabetol..

[33] Blanco M., Hernández M.T., Strauss K.W., Amaya M. (2013). Prevalence and risk factors of lipohypertrophy in insulin-injecting patients with diabetes. Diabetes Metab..

[34] Danne T., Phillip M., Buckingham B.A., Jarosz-Chobot P., Saboo B., Urakami T., Battelino T., Hanas R., Codner E. (2018). ISPAD Clinical Practice Consensus Guidelines 2018: Insulin treatment in children and adolescents with diabetes. Pediatr. Diabetes.

[35] Al Hayek A.A., Robert A.A., Braham R.B., Al Dawish M.A. (2016). Frequency of Lipohypertrophy and Associated Risk Factors in Young Patients with Type 1 Diabetes: A Cross-Sectional Study. Diabetes Ther..

[36] Demir G., Er E., Atik Aktınok Y., Özen S., Darcan Ş., Gökşen D. (2021). Local complications of insulin administration sites and effect on diabetes management. J. Clin. Nurs..

[37] Johansson U.B., Amsberg S., Hannerz L., Wredling R., Adamson U., Arnqvist H.J., Lins P.E. (2005). Impaired absorption of insulin aspart from lipohypertrophic injection sites. Diabetes Care.

[38] De Coninck C., Frid A., Gaspar R., Hicks D., Hirsch L., Kreugel G., Liersch J., Letondeur C., Sauvanet J.P., Tubiana N. (2010). Results and analysis of the 2008–2009 Insulin Injection Technique Questionnaire survey. J. Diabetes.

[39] Piona C., Ventrici C., Marcovecchio L., Chiarelli F., Maffeis C., Bonfanti R., Rabbone I. (2021). Long-term complications of type 1 diabetes: What do we know and what do we need to understand?. Minerva Pediatr..

[40] Šoupal J., Škrha Jr J., Fajmon M., Horová E., Mráz M., Škrha J., Prázný M. (2014). Glycemic variability is higher in type 1 diabetes patients with microvascular complications irrespective of glycemic control. Diabetes Technol. Ther..

